# Limited, episodic diversification and contrasting phylogeography in a New Zealand cicada radiation

**DOI:** 10.1186/1471-2148-12-177

**Published:** 2012-09-11

**Authors:** David C Marshall, Kathy B R Hill, Katharine A Marske, Colleen Chambers, Thomas R Buckley, Chris Simon

**Affiliations:** 1 Department of Ecology and Evolutionary Biology, University of Connecticut, 75 N. Eagleville Rd, Storrs, CT, 06269, USA; 2 Center for Macroecology, Evolution and Climate, University of Copenhagen, Copenhagen, Denmark; 3 Landcare Research, Private Bag 92170, Auckland, New Zealand; 4 School of Biological Sciences, The University of Auckland, Private Bag 92019, Auckland, New Zealand; 5 Allan Wilson Centre for Molecular Ecology and Evolution, Auckland, New Zealand; 6 School of Biological Sciences, Victoria University of Wellington, Wellington, New Zealand

## Abstract

**Background:**

The New Zealand (NZ) cicada fauna contains two co-distributed lineages that independently colonized the isolated continental fragment in the Miocene. One extensively studied lineage includes 90% of the extant species (*Kikihia* + *Maoricicada* + *Rhodopsalta*; ca 51 spp.), while the other contains just four extant species (*Amphipsalta* – 3 spp. + *Notopsalta* – 1 sp.) and has been little studied. We examined mitochondrial and nuclear-gene phylogenies and phylogeography, Bayesian relaxed-clock divergence timing (incorporating literature-based uncertainty of molecular clock estimates) and ecological niche models of the species from the smaller radiation.

**Results:**

Mitochondrial and nuclear-gene trees supported the monophyly of *Amphipsalta*. Most interspecific diversification within *Amphipsalta*-*Notopsalta* occurred from the mid-Miocene to the Pliocene. However, interspecific divergence time estimates had large confidence intervals and were highly dependent on the assumed tree prior, and comparisons of uncorrected and patristic distances suggested difficulty in estimation of branch lengths. In contrast, intraspecific divergence times varied little across analyses, and all appear to have occurred during the Pleistocene. Two large-bodied forest taxa (*A. cingulata, A. zelandica*) showed minimal phylogeographic structure, with intraspecific diversification dating to ca. 0.16 and 0.37 Ma, respectively. Mid-Pleistocene-age phylogeographic structure was found within two smaller-bodied species (*A. strepitans* – 1.16 Ma, *N. sericea* – 1.36 Ma] inhabiting dry open habitats. Branches separating independently evolving species were long compared to intraspecific branches. Ecological niche models hindcast to the Last Glacial Maximum (LGM) matched expectations from the genetic datasets for *A. zelandica* and *A. strepitans*, suggesting that the range of *A. zelandica* was greatly reduced while *A. strepitans* refugia were more extensive. However, no LGM habitat could be reconstructed for *A. cingulata* and *N. sericea*, suggesting survival in microhabitats not detectable with our downscaled climate data.

**Conclusions:**

Unlike the large and continuous diversification exhibited by the *Kikihia-Maoricicada-Rhodopsalta* clade, the contemporaneous *Amphipsalta-Notopsalta* lineage contains four comparatively old (early branching) species that show only recent diversification. This indicates either a long period of stasis with no speciation, or one or more bouts of extinction that have pruned the radiation. Within *Amphipsalta-Notopsalta*, greater population structure is found in dry-open-habitat species versus forest specialists. We attribute this difference to the fact that NZ lowland forests were repeatedly reduced in extent during glacial periods, while steep, open habitats likely became more available during late Pleistocene uplift.

## Background

**Figure 1:**
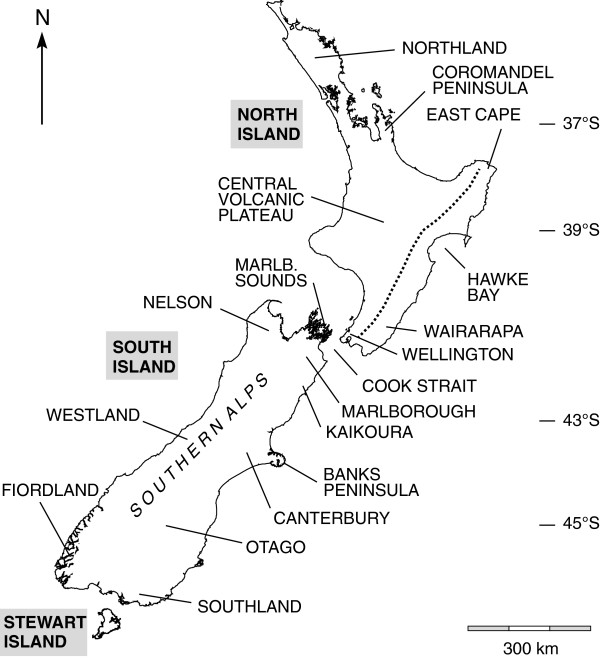
**The mainland New Zealand islands showing major locations referenced in the text.** The dotted line in eastern NI approximates the major SW-NE mountain axis (which lies on the Alpine Fault together with the Southern Alps in SI). The latitudinal reach of the mainland islands extends from approximately 34° S to 47° S.

**Figure 2:**
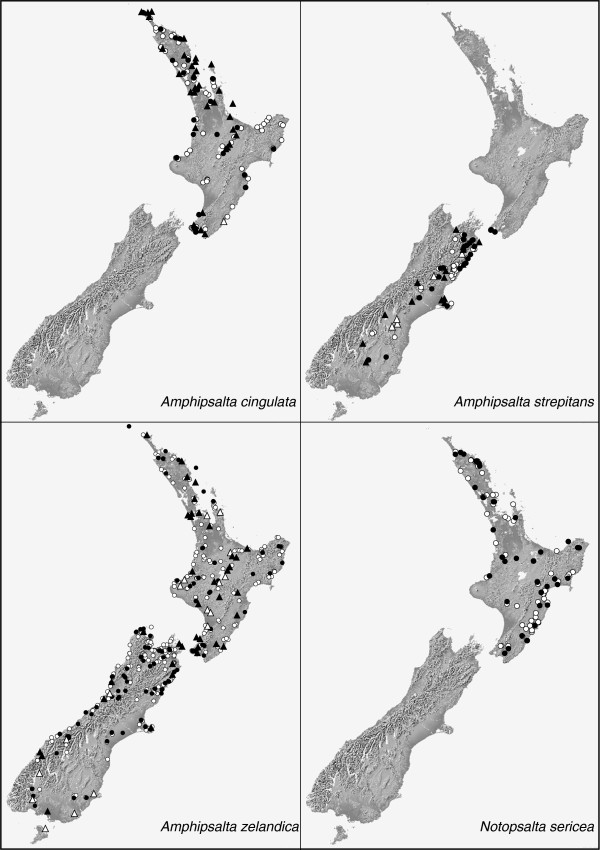
**Distributions of the *Amphipsalta *and *Notopsalta *species.** Records are based on C. Simon lab (circles) and Dugdale and Fleming [11] (triangles) observations, plotted on a shaded elevation map. Filled symbols indicate collected specimens; open symbols represent song records only.

The cicadas of New Zealand (NZ) present an example of rapid ecological radiation in response to landscape and climate changes, mainly of the Pliocene (5.3 - 2.6 Ma) and Pleistocene (2.6 - 0.1 Ma) epochs [1-3]. Approximately 55 NZ species form two monophyletic groups, each derived from an overwater colonization event [4,5]. Most belong to a large clade containing three genera: *Kikihia* Dugdale (ca. 28 taxa), *Maoricicada* Dugdale (ca. 20 taxa), and *Rhodopsalta* Dugdale (3 taxa), which are related to New Caledonian cicadas [4,5]. Species of this clade are distributed across all three main islands (North, South, Stewart; see Figure 1 and Figure 2) and several outer islands, and together they inhabit a variety of low- and mid-elevation habitats [6-9]. The diversification of *Maoricicada*, in particular, is an extraordinary case for the family Cicadidae because its species are largely alpine in distribution. Molecular-clock studies suggest that diversification in both *Kikihia* and *Maoricicada* began around 6–7 Ma and accelerated through the Pliocene as mountain-building increased temperature and moisture contrasts throughout NZ and isolated populations geographically [1,2,10].

The remaining NZ cicada species (two genera) belong to the same tribe (Cicadettini) but form a separate monophyletic clade related to Australian taxa [4,5]. This clade is also widely distributed across NZ and includes the familiar wing-clapping *Amphipsalta* Fleming species. However, these species have received comparatively little study. *Amphipsalta zelandica* (Boisduval) inhabits low- to mid-elevation forests throughout mainland NZ and nearby islands, while *A. cingulata* (Fabricius) is found only on North Island (NI), mainly in trees of coastal scrub habitats [11] (Figure 2). *A. strepitans* (Kirkaldy) utilizes shrubs (*Discaria*, *Cassinia* – [11]) in dry, open habitat, and it sings on both vegetation and rocks [12,13] from southern NI to southeastern South Island (SI). *Notopsalta sericea* (Walker) sings from vegetation, volcanic rocks, and clay banks in steep, open country [13] throughout NI. The small size of the *AmphipsaltaNotopsalta* radiation is remarkable because its ancestor arrived in NZ at approximately the same time as the *KikihiaMaoricicadaRhodopsalta* ancestor, and because it likely experienced similar Miocene and Pleistocene landscape and climate changes [14,15].

In this study we use multi-gene molecular-phylogenetic methods, divergence-time analysis, intraspecific sampling, and ecological niche models (ENMs) to examine the evolution of *Amphipsalta*-*Notopsalta*. We find that few extant lineages in the clade originated during the late Miocene and Pliocene, either because speciation rates were then low or because most lineages appearing during that time later went extinct. In addition, we find evidence of Pleistocene-age intraspecific diversification with contrasting phylogeographic histories across the species. Species’ modeled geographic ranges at the LGM (Last Glacial Maximum, c. 22,000 cal yrs BP) varied from distributions similar to at present to entirely absent at the LGM refugia.

## Results

### Maximum-likelihood phylogenetic and phylogeographic relationships

All sequences were submitted to GenBank under accession numbers JX675238 - JX675427 (COI), JX675449 - JX675465 (Calmodulin), and JX675428 - JX675448Â (EF-1α). Character profiles and partition-specific models selected are shown in Table 1. The two nuclear loci were characterized by low numbers of parsimony-informative sites among ingroup taxa. The absence of a gamma parameter in the Modeltest output for those genes indicates either a simple pattern of among-site rate variation, or insufficient data for estimation of a more complex model. PartitionFinder identified support for a three-partition mtDNA model (with each codon position modeled separately).

**Table 1:** Nuclear and mtDNA dataset statistics, with outgroup excluded, and including partition-specific substitution models from Modeltest; asterisked partitions were used for the final analyses

**Gene/partition**	**# Sites**	**# Variable sites**	**# Parsimony informative**	**Model selected**
COI (1^st^ pos.)*	451	21	16	TrN + I
COI (2^nd^ pos.)*	451	5	4	F81
COI (1^st^ + 2^nd^ pos.)	902	26	20	HKY + G
COI (3^rd^ pos.)*	451	203	184	TIM + G
COI (all sites)	1353	229	204	GTR + I + G
Calmodulin	719	44	29	TIM + I
EF-1a	1304	69	56	HKY + I

**Figure 3:**
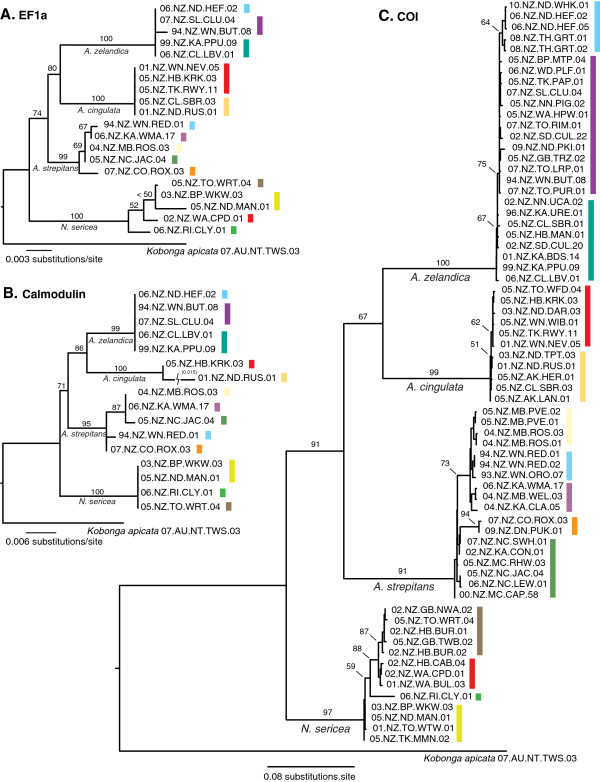
**Maximum-likelihood gene trees estimated in Garli v2.0.** Bootstrap support values from 100 pseudoreplicates are shown on major branches. Colors indicate membership in groups shown on Figure 5.

COI sequences of the four species formed strongly supported monophyletic groups, and *Amphipsalta* was recovered as a monophyletic group (91% ML bootstrap), but *A. zelandica* and *A. cingulata* were only weakly supported as sister-taxa (Figure 3). COI patristic genetic distances between the four species ranged from 0.21-0.31 substitutions/site (uncorrected *p* distance 0.061-0.082). The nuclear-gene trees agreed with each other and with the COI topology, and they also supported a monophyletic *Amphipsalta* (>70%). *A. cingulata* and *A. zelandica* were supported as sister-species with >70% ML bootstrap support in both nuclear gene trees (Figure 3).

Phylogeographic structure was evident within all species, but with considerable differences in levels of divergence. Little intraspecific divergence (weak structure) in COI was detected for either *A. cingulata* or *A. zelandica*. Maximum patristic genetic distances between haplotypes from the partitioned ML analysis (Figure 3) were 0.005 substitutions/site (s/s) in *A. cingulata* (0.004 uncorrected, or *p* distance) and 0.011 s/s (0.009 uncorrected) in *A. zelandica*. For both species, haplotype patterns suggested a north–south phylogeographic break within NI, especially in *A. zelandica*, where a distinctive haplotype cluster was limited to northern NI (ML bootstrap 64%).

ML patristic distances within *A. strepitans* and *N. sericea* were 3–6 times greater, with obvious geographical patterning (Figure 3). Within *A. strepitans*, haplotypes from southern SI differed by up to 0.04 s/s (0.024 *p* distance) from haplotypes in central NZ. The lone *N. sericea* haplotype from southwestern NI differed from other *N. sericea* haplotypes by 0.038 s/s in COI (0.025 *p* distance). *N. sericea* haplotypes along the eastern coast formed geographically coherent sister-clades, and haplotypes from the northern NI were nearly genetically identical (Figure 3). Otherwise, the phylogenetic relationships between regional haplotype clusters were poorly resolved in both *N. sericea* and *A. strepitans*.

The comparatively limited EF-1α dataset mirrored some intraspecific patterns observed in COI. EF-1α sequences for *A. zelandica* and *A. cingulata* were nearly identical within species, as for COI. Within *A. strepitans*, the central Otago sequence was divergent from the rest, and the Wellington (southern NI) and Marlborough (northern SI) samples were sister to one another. The *N. sericea* EF-1α sequences yielded the same three-way split observed between southwestern, eastern, and northern NI.

Intraspecific relationships observed in the calmodulin dataset generally did not match those found in COI and EF-1α. Notably, no sequence divergence was observed between the four *N. sericea* specimens even though these individuals were highly divergent at the other two loci. Two divergent haplotypes (mostly differing at a gene section containing tandem repeats) were found for *A. cingulata,* but little genetic divergence for COI or EF-1α was observed for individuals possessing those haplotypes. Differences in phylogenetic signal between calmodulin and EF-1α/mtDNA have been detected in other NZ cicada species, with calmodulin divergence rates much higher for some species than for their congeners [16].

### Intraspecific diversification

The GMYC algorithm identified independent lineages within each of the four described species (Additional file 1: Figure S1) – six in *Amphipsalta zelandica*, two in *A. cingulata*, seven in *A. strepitans*, and four in *Notopsalta sericea*; the null hypothesis was rejected by a likelihood-ratio test (null likelihood = 238.2016; GMYC likelihood = 246.8606; *P* < 0.006). However, results were dependent on the dataset used. When *A. zelandica* and *A. cingulata* haplotypes were removed to see if their low intraspecific divergence was pulling the between/within-species threshold closer to the present, the GMYC algorithm diagnosed nearly every haplotype of the remaining species as an independent population lineage, although the results were only marginally significant (null likelihood = 141.2776, GMYC likelihood = 145.5174, *P* < 0.037). AMOVA results (Table 2) indicated considerable geographic-genetic structure, with greater than 30% of the genetic variance explained at the regional level in each species. *Amphipsalta strepitans* and *N. sericea* exhibited the highest levels of genetic variance between regions.

**Table 2:** **Analysis of molecular variance (AMOVA) showing partitioning of mtDNA genetic variation within and among regions**^**1**^

**Source of variation**	**d.f.**	**Sum of squares**	**Variance components**	**Percentage of variation**
*A. cingulata*	F_st_ = 1.00000			
Among regions	2	10.371	0.47420	34.35*
Among populations within regions	17	18.829	0.90615	65.65*
Within populations	5	0.000	0.00000	0.00*
Total	24	29.200	1.38035	
*A. zelandica*	F_st_ = 0.53778			
Among regions	5	50.856	0.49576	28.74*
Among populations within regions	69	98.313	0.43187	25.04*
Within populations	37	29.500	0.79730	46.22*
Total	111	178.670	1.72492	
*A. strepitans*	F_st_ = 0.98929			
Among regions	3	100.958	4.20935	40.56*
Among populations within regions	11	100.167	6.05782	58.37*
Within populations	9	1.000	0.11111	1.07*
Total	23	202.125	10.37829	
*N. sericea*	F_st_ = 0.83766			
Among regions	2	54.042	2.51745	40.87*
Among populations within regions	32	87.958	2.64237	42.90
Within populations	2	2.000	1.00000	16.23
Total	27			

^1^ Regions were defined by a six-way, north-to-south partition of the NZ mainland. Asterisks indicate statistically significant tests (*p* < 0.05).

### Pairwise genetic divergence plots

**Figure 4:**
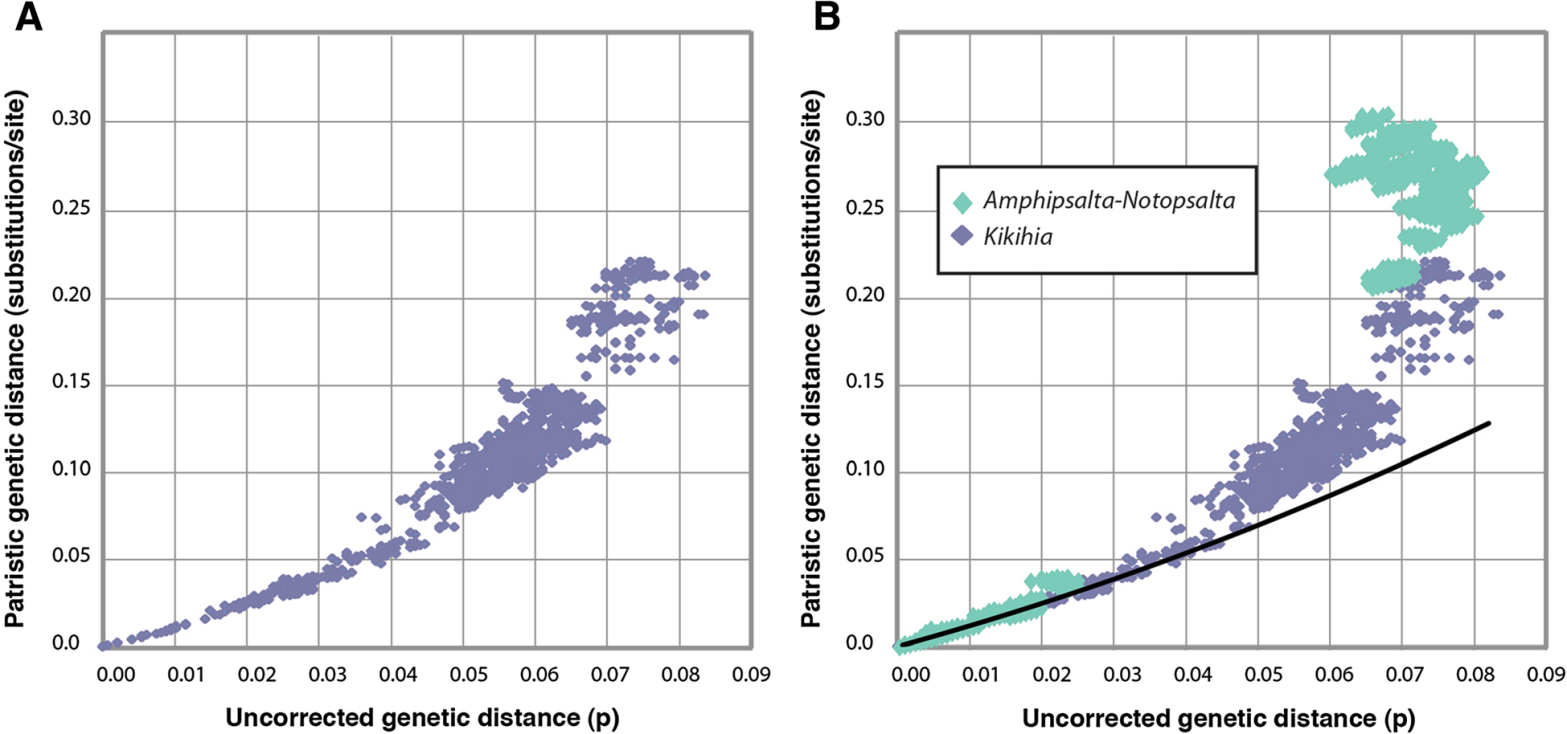
**Plots of mtDNA uncorrected distance vs. ML patristic distance.A**) Plot from a well-sampled *Kikihia* COI + COII mtDNA ingroup dataset containing 30 species [1]. **B**) Plot from the *AmphipsaltaNotopsalta* COI dataset from this study (light green dots), superimposed on the plot from A. The datasets span a nearly identical range of uncorrected distance values, but many corrected pairwise distances are over twice as large for *AmphipsaltaNotopsalta* at uncorrected distances > 6%. The trendline in B was fitted to the *Kikihia* data points under 4% uncorrected distance

The relationships between corrected (patristic) and uncorrected mtDNA distances for *Amphipsalta-Notopsalta* (this study) and for *Kikihia* COI + COII sequences [1] were similar only at low genetic distances (Figure 4). For both datasets, this relationship was curved upward only slightly at low uncorrected genetic distances. At greater distances, the relationships diverged. Despite the fact that maximum uncorrected divergence levels were similar for the two datasets (ca. 8%), the maximum patristic distances were 0.22 s/s in *Kikihia* and 0.31 s/s in *Amphipsalta-Notopsalta* (Figure 4). The *Amphipsalta-Notopsalta* curve was notably ragged at high genetic distances, with substantially different patristic distances reconstructed for some taxa with uncorrected distances greater than 6%.

### Divergence-time analysis

The BEAST analyses under different tree models showed a strong effect of the prior on interspecific divergence times, but comparatively little effect on intraspecific haplotype divergences and species’ MRCA ages (Table 3). The birth-death model resulted in a mean root age of 24.45 Ma, compared to only 4.82 Ma for the Yule and approximately 17 Ma for each of the two coalescent models. Intraspecific divergences were dated to the Pleistocene in all cases. Analyses run with no data in order to estimate the prior returned divergence times close to zero for all nodes.

**Table 3:** Mean divergence-time estimates (in Ma) and 95% confidence intervals from BEAST analyses using different tree models

**Tree model**	**root**	***Amphipsalta-Notopsalta***	***Amphipsalta***	***N. sericea *MRCA**	***A. strepitans *MRCA**	***A. zelandica *MRCA**	***A. cingulata *MRCA**
Yule	4.82 *(3.87-5.69)*	3.70 *(2.95-4.49)*	2.97 *(2.42-3.56)*	1.01 *(0.64-1.39)*	0.84 *(0.60-1.11)*	0.39 *(0.26-0.54)*	0.23 *(0.13-0.34)*
birth-death	24.45 *(14.86-38.10)*	12.59 *(7.6-17.2)*	9.22 *(5.75-12.82)*	1.36 *(0.70-2.06)*	1.16 *(0.66-1.70)*	0.37 *(0.19-0.56)*	0.16 *(0.06-0.27)*
coalescent (constant)	17.43 *(11.11-23.97)*	8.21 *(5.67-10.69)*	5.91 *(4.04-7.61)*	1.28 *(0.75-1.87)*	1.07 *(0.65-1.44)*	0.39 *(0.22-0.59)*	0.18 *(0.08-0.28)*
coalescent (expansion)	17.00 *(10.70-23.42)*	8.13 *(5.64-10.35)*	5.85 *(4.20-7.59)*	1.26 *(0.77-1.89)*	1.07 *(0.68-1.52)*	0.39 *(0.23-0.59)*	0.18 *(0.08-0.30)*

Estimates of interspecific divergence times were strongly influenced by the tree prior, while ages of species’ MRCAs and intraspecific divergences varied much less.

**Figure 5:**
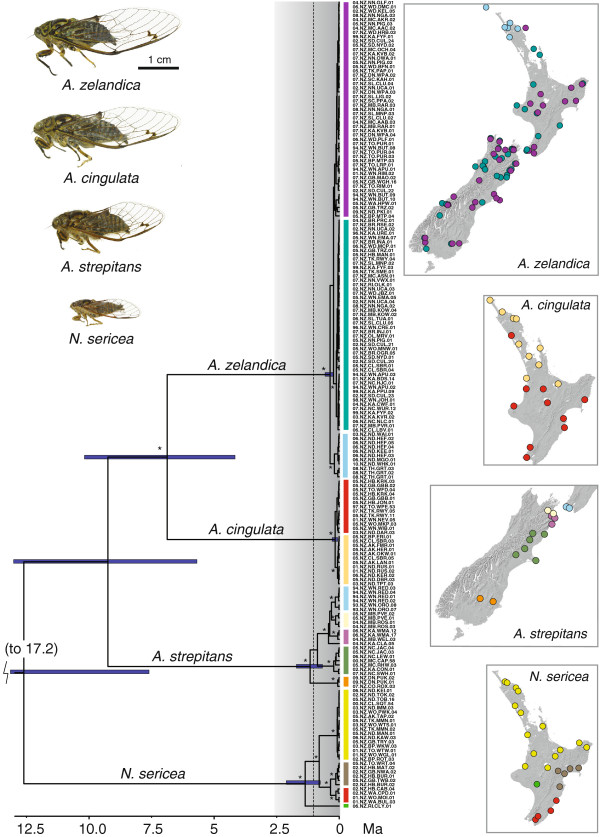
**BEAST v 1.6.1 relaxed-clock chronogram (maximum clade compatibility tree, with mean node ages calculated), with outgroup taxon and branches pruned.** Tree was constructed using the *Amphipsalta*-*Notopsalta* mtDNA COI dataset and calibrated using literature estimates of per lineage molecular clock rates in insects. Asterisks indicate major branches with > 90% posterior probability. Horizontal blue bars indicate 95% credible intervals on the mean divergence time estimates. Major haplotype groups are shown, by color, on the accompanying maps of the NZ landscape. Shading represents climatic deterioration during the Pleistocene, and the vertical dotted line represents accelerated uplift in northern NZ around 1 Ma.

While the estimated marginal likelihoods were similar for the birth-death, constant-size coalescent, and expansion-growth coalescent models, the 2 ln Bayes factor score indicated support for the birth-death model (Table 4). Under this model, the *Amphipsalta* + *Notopsalta* ancestor diverged from the outgroup approximately 24 Ma, and *Notopsalta* split from the *Amphipsalta* common ancestor at around 12.5 Ma. The remaining interspecific divergences were dated to ca. 9 Ma (*A. strepitans*) and 7 Ma (*A. cingulata* – *A.zelandica* split) (Figure 5). The earliest splits within *A. strepitans* and *N. sericea* dated to the mid-Pleistocene (ca. 1.2 Ma and 1.4 Ma, respectively), while splits within *A. zelandica* and *A. cingulata* dated to the Late Pleistocene (mean ca. 0.4 and 0.2 Ma, respectively). Credible intervals for the intraspecific coalescences were similar in magnitude (relative to the mean estimates) to those observed for the interspecific splits (Table 3). The *meanrate* estimate (0.012 s/s), reflecting the mean clock rate across the tree, remained close to the fixed *ucld.mean* rate, as expected because no calibrations other than the fixed values for *ucld.mean* and *ucld.stdev* were used in the analysis. Because we fixed *ucld.stdev*, we could not use this statistic to test for clocklike evolution, but because clocklike data can be accommodated by a relaxed clock model our approach is conservative.

**Table 4:** Bayes factor scores (from Tracer v1.5) from comparisons of alternative tree models for the BEAST divergence time analyses

**Tree model**	**estimated mean -lnL**	**S. E.**	**2 (ln Bayes factor score)**			
			**vs. Yule**	**vs. birth-death**	**vs. coalescent (constant size)**	**vs. coalescent (expansion)**
Yule	−4236.94	±2.085	–	−179.978	−160.334	−157.378
birth-death	−4146.95	±1.191	179.978	–	19.644	22.602
coalescent (constant size)	−4156.77	±1.113	160.334	−19.644	–	2.956
coalescent (expansion)	−4158.25	±0.912	157.378	−22.602	−2.956	–

Strong support for the birth-death model was indicated, according to the criterion 2 ln Bayes factor > 10.

The maximum clade compatibility tree from the BEAST analysis (Figure 5) found different intraspecific root positions for *N. sericea* and *A. strepitans*, compared to the ML results. As a result, some geographically coherent haplotype clades that appeared paraphyletic in the ML tree were found to be monophyletic with strong support (> 90% posterior probability) in the BEAST analysis.

### Ecological niche models

**Figure 6:**
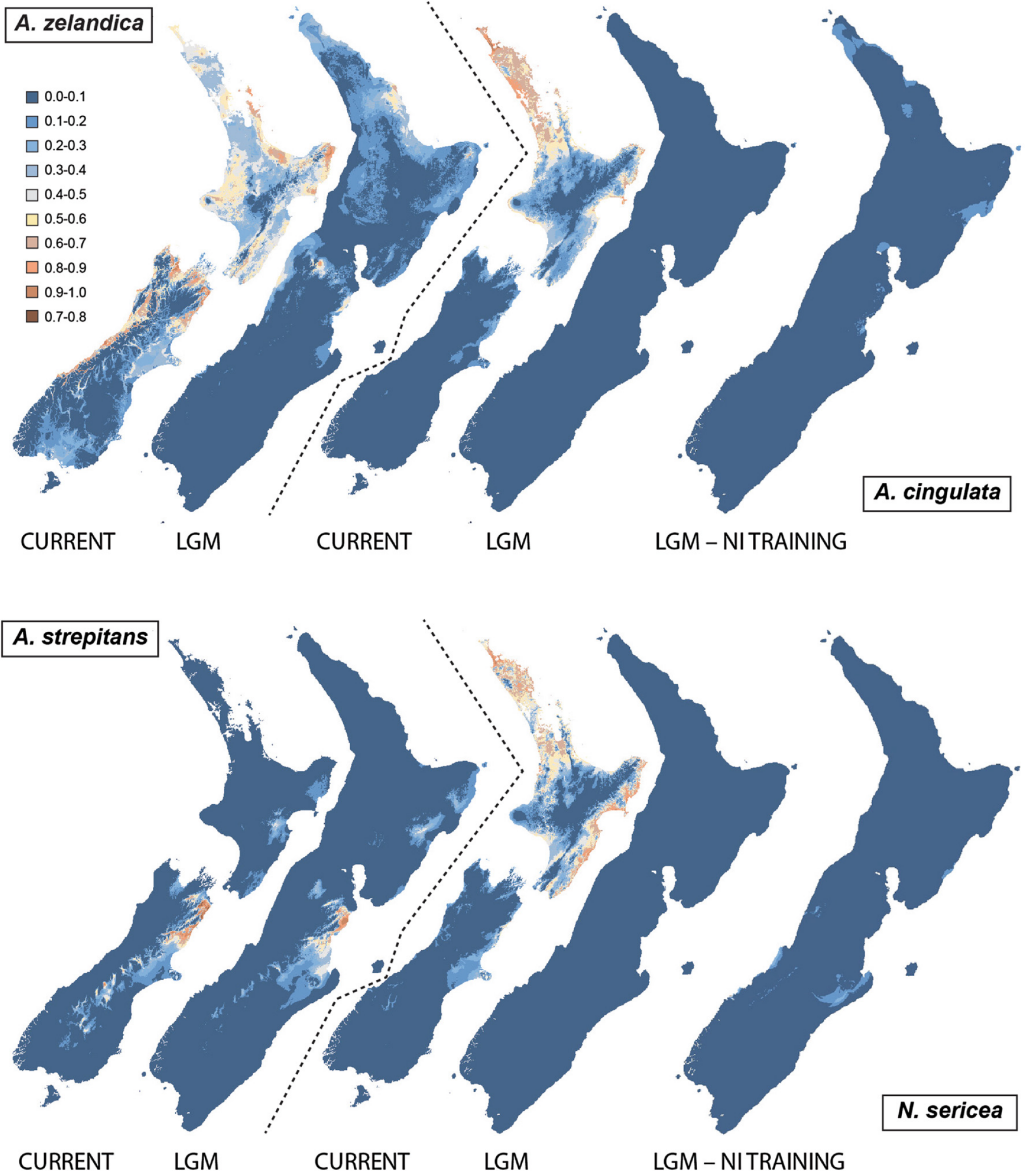
**Projected current and Last Glacial Maximum distributions for each *Amphipsalta *and *Notopsalta *species, modeled in Maxent.** For *A. cingulata* and *N. sericea*, which are found only on NI, two projected LGM distributions are shown – one with the model trained on background localities from all of NZ, and the other trained only on NI localities. Scale shows climate suitability values; warmer colors indicate higher suitability.

Bioclimatic models of the distribution of *Amphipsalta zelandica* yielded an average AUC of 0.806 (s.d. 0.036) in the 10-fold cross-validation runs and performed significantly better than random (*P* < 0.05) in the binomial omission tests across all thresholds and runs. Heuristic estimation of relative contributions of the environmental variables indicated that annual temperature (38.9%) and February temperature (20.5%) were the top predictors of the distribution of *A. zelandica*. Effects of clamping during LGM projection were minor and restricted to montane areas. Under current climate conditions, A. *zelandica* was projected to be widely distributed throughout NI and northern SI in lowland regions (Figure 6). During the LGM, most of NZ was projected as less suitable for *A. zelandica*, with the exception of the Coromandel Peninsula and East Cape in northern NI and Karamea, Central Nelson and Kaikoura in northern SI (Figure 6).

Models for the distribution of *A. strepitans* also performed significantly better than random in the binomial omission tests in all but one run and yielded an average AUC of 0.940 (s.d. 0.054) (note that AUC values tend to be higher for geographically restricted taxa). The top predictor of the distribution of *A. strepitans* was October vapor pressure deficit (87.1%), with February temperature a distant second (4.4%). Effects from clamping during LGM projection were largely restricted to the central Southern Alps. The species’ projected distribution centered on the northeastern SI, particularly the coastal Kaikoura Ranges (Figure 6), under both climate scenarios, and the projected LGM distribution was slightly larger (more area with suitability >0.5) than that for current conditions. Notably, the model failed to capture the southern extent of the species’ range – several sampled populations fall within the region of 0-10% probability predicted by Maxent. As this may have been due to the high number of localities from the northern SI, resulting in over-fitting of regional conditions, input localities for *A. strepitans* were thinned to a minimum distance of 5 km apart (removing 15 localities from the northern SI) and modeled again as above. The resulting models were no better at capturing the southern extent of the species’ distribution (data not shown), which is represented by only 5 localities south of central Canterbury.

Results for *Notopsalta sericea* were also significantly better than random, with only one threshold for one run yielding *P* > 0.05 in the binomial omission tests, and an average AUC of 0.874 (s.d. 0.031). Top climatic predictors were February temperature (55.9%) and minimum temperature (23.8%), and no effects from clamping were detected during projection. Under modern conditions, northern and coastal NI were projected as climatically suitable, but none of NZ was projected to contain suitable conditions during the LGM (Figure 6).

Models for *A. cingulata* yielded similar geographic projections, with much of lowland NI projected as climatically suitable under modern conditions, and no refugia projected for the LGM (Figure 6). Results from the binomial omission tests were significantly different from random except for one data partition which yielded multiple thresholds with *P* > 0.5. The average AUC across ten runs was 0.881 (s.d. 0.043), and annual temperature (73.2%) and minimum temperature (11.0%) were the top predictors of the distribution of *A. cingulata*.

When model calibration for *N. sericea* and *A. cingulata* was restricted to environmental conditions from NI, performance markedly declined, yielding average AUCs of 0.732 (s.d. 0.089) and 0.727 (s.d. 0.102), respectively, and multiple binomial omission tests for multiple runs yielded *P* > 0.05. Areas of uncertainty (clamping) appeared when projecting onto the full LGM surface, and once these areas were removed, no likely refugia were detected, although some regions had climatic suitability above zero (Figure 6).

## Discussion

### Molecular clocks and divergence-time estimation for the *Amphipsalta*-*Notopsalta* radiation

#### Molecular clock calibration and substitution models

Insect molecular-clock studies often apply a mtDNA calibration of 2.3% pairwise distance per my, or 0.0115 substitutions/site/my/lineage, derived by Brower [17]. However, two estimates used by Brower were revised downward during re-analysis [18-20], and the remaining data were derived from rRNA, DNA-DNA hybridization, or RFLP datasets, so their applicability to mtDNA protein coding genes such as COI is unclear. In addition, none of the data used in the Brower study were corrected for multiple hits with data partitioning or the use of gamma-distributed rates, as most data are today [21-24]. If studies that calibrate molecular clocks do not correct for multiple hits as thoroughly as the analyses that employ them, the date estimates will be biased [25].

The Bayesian relaxed-clock approach in BEAST allows the investigator to conservatively accommodate a range of plausible COI clock values with a single prior probability distribution in each analysis. We calibrated our analyses with fixed values for the mean branch rate and the standard deviation of that mean across the tree (*ucld.mean* and *ucld.stdev* parameters), set such that the 95% credible intervals included the published rates cited in Table 5.

**Table 5:** Published arthropod substitution rate estimates for the mitochondrial gene cytochrome oxidase subunit I (COI)

**Reference**	**Taxon**	**Corrected pairwise distance (%)**	**Per lineage rate, (subst./site/my)**
Caccone and Sbordoni [95]	*Ovobathysciola* cave beetles	1.2	0.006
Caccone and Sbordoni [95]	*Speonornus* cave beetles	1.3	0.0065
Farrell [19]	*Tetraopes* beetles	1.5	0.0075
Juan et al. [96]	*Pimelia* beetles, Canary Is.	1.3	0.0065
Knowlton and Weigt [20], Morrison et al. [97]	*Alpheus* snapping shrimp	1.4	0.007
Papadopoulou et al. [25]	Tenebrionid beetle genera (6)	3.54	0.0177
Schubart et al. [98]	*Sesarma* land crabs #1	1.28, 1.9	0.0064, 0.0095
Sota and Hayashi [85]	*Plateumaris* leaf beetles	1.6	0.008

Although these distances were reported as percentages in the papers referenced, a common practice, this is not ideal for corrected distances since the values can exceed 100%.

The divergence-time and ML analyses in this study may have suffered from poor branch-length estimation (with “branch lengths” estimated as rate x time products in the case of BEAST), perhaps related to the limited number of deep splits in the tree. Results from the BEAST analyses, specifically the ages of the interspecific splits, were strongly influenced by the choice of tree prior (Table 3). Plots of corrected vs. patristic distances in the *Amphipsalta-Notopsalta* and published *Kikihia* datasets showed that corrected distances were considerably greater for *Amphipsalta*-*Notopsalta* at the deepest levels examined, although both plots initially followed similar trajectories (Figure 4). Although the two datasets may have evolved under different mechanisms, the magnitude of the difference and the ragged nature of the *Amphipsalta*-*Notopsalta* plot are causes for concern. Further examination of the relationships between tree shape, dataset information, and branch lengths in maximum-likelihood and divergence-time analyses would likely prove useful. Notably, the confidence intervals on the intraspecific data estimates from BEAST were much narrower and largely unaffected by the tree prior.

#### Interspecific vs. intraspecific divergence timing

Unlike the NZ *Kikihia-Maoricicada-Rhodopsalta* cicada radiation, the *Amphipsalta-Notopsalta* radiation contains no described species with Pleistocene origins. An earlier genus-level phylogenetic analysis including *A. cingulata* and *N. sericea* demonstrated a high level of genetic divergence between these species, consistent with a late Miocene to early Pliocene split (ca. 5–6 Ma) [5]. We find here that all interspecific divergences in the clade predate the Pleistocene (i.e., > 2.6 Ma), although these dates are uncertain. In addition, the low number of species in the *AmphipsaltaNotopsalta* radiation is not due to more recent diversification, since the earliest divergences of both clades appear to have occurred in the mid-Miocene [2,5]. (Due to extensive collecting over multiple decades, undiscovered extant species are unlikely.)

Although species-level diversification apparently preceded the Pleistocene, the genetic data suggest that several independently evolving, morphologically cryptic populations have formed during that period. All four species contain mtDNA phylogeographic structure, i.e., unique haplotypes or haplotype clusters occupying exclusive geographic ranges, with uncorrected genetic distances between clades approaching 2.5%. The nuclear data show some concordant patterns, but they do not confirm all of the mtDNA clades, and there is conflict in some cases (e.g., all four *N. sericea* individuals have the same calmodulin allele, despite considerable mtDNA structure). Nuclear genes tend to evolve much more slowly than mtDNA, so considerable structure can appear in mitochondrial genes before concurrent changes occur in nDNA. Furthermore, the larger effective population sizes of nuclear genes mean that more time is needed for lineage sorting to become complete [26], so the lack of detailed structure in calmodulin and EF-1α does not necessarily mean that the mtDNA clades are freely exchanging such genes. However, nuclear genes can also reveal sex-specific gene flow patterns that a uniparental marker like mtDNA can miss. The phylogeographic evidence of intraspecific diversification helps to alleviate the potential concern that, by sampling extensively “within species” for the BEAST analyses, we may be overestimating intraspecific sequence divergence and therefore the MRCA ages of the four species [27].

In NZ *Kikihia* and *Maoricicada*[16,28-30], mtDNA phylogeography and song patterns indicate Pleistocene-age allopatric diversification within currently recognized species. Populations with greater than 2.5% uncorrected mtDNA divergence frequently, although not always, exhibit discrete differences in song [16,29-31] – and in some cases conflicting patterns in EF-1α. The largest intraspecific mtDNA genetic distances within *A. strepitans* and *N. sericea* approach this threshold. We have not observed regional differences in the songs of *Amphipsalta* and *Notopsalta* during field collection and observation, but detailed examination might uncover minor variation concordant with the mtDNA tree.

The GMYC analysis (Additional file 1: Figure S1), which was intended to identify the shift from between- to within-population branching patterns [32], yielded results consistent with the inference that some of the geographically coherent mtDNA phylogroups are independently evolving populations. However, the large number of clades inferred for *A. zelandica* is surprising give the low sequence divergence within that species, and the GMYC results changed substantially when only *N. sericea* and *A. strepitans* haplotypes were examined. It is possible that the marginal statistical significance observed in the latter test is a sign of too little data for effective estimation of branching-rate shifts despite the likely existence of several independently evolving populations within these taxa.

If the mtDNA clades represent independently evolving subspecific populations, then at least 2–5 allopatric population-lineages per species have formed in *AmphipsaltaNotopsalta* during the Pleistocene, a rapid rate compared to the Late Miocene and Pliocene. This contrasts with the results from *Kikihia*, where the rate of population-lineage formation has remained approximately constant since the early Pliocene [1].

### Potential drivers of intraspecific divergence in *Amphipsalta-Notopsalta*

#### Limited phylogeographic structure in forest taxa

The low level of intraspecific genetic divergence characterizing *Amphipsalta cingulata* and *A. zelandica* is consistent with their dependence on temperate forest habitat. NZ forests progressively declined in extent throughout the Pleistocene, especially during the last million years. During the LGM (~22 ka), the principal forest refugia were located in northern NI and/or northwestern SI [33-35], with smaller fragments located farther south [36]. Roughly similar regions were predicted for *A. zelandica* refugia (*A. cingulata* refugia were not successfully reconstructed). Both *A. cingulata* and *A. zelandica* contain haplotype clusters consistent with survival during the LGM in as few as two locations, one more northern and one more southern [37]. However, the phylogeographic structure observed within *A. zelandica* is insufficiently detailed to confirm the ENM predictions of two major refugia in NI and 2–3 in SI, although the genetic data are consistent with the absence of refugia in central or southern SI.

The lack of structure within *A. zelandica* is remarkable when compared with phylogenetic patterns observed in other forest species believed to have survived in SI [31,38-40]. While NZ forest taxa often show evidence of geographic restriction during the LGM, different species exhibit phylogeographic variation that likely relates to differences in life-history parameters or habitat specialization. For example, forest taxa including beetles [39-41] and a fern [42] appear to have survived glacial cold phases in small forest fragments in southern NZ and retained considerable phylogeographic structure there. In contrast, the only other widespread NZ forest cicada, *Kikihia subalpina,* retains considerable geographic structure on NI (where it lives mainly in subalpine scrub), but limited structure on SI where it inhabits lowland forest [31]. Greater Pleistocene stability of NI habitats has been linked to persistence of beetle species (as inferred from fossils; [43]). It is possible that, for cicadas, additional factors are needed to explain why the forest taxa have been unable to survive in southern NZ during colder climate phases (see also [41]).

Because the intraspecific lineages within *A. zelandica* and *A. cingulata* were inferred to coalesce around 370 ky and 160 ky, respectively, both species were likely limited to a single refugium during the Late Pleistocene. Some support for this idea is observed in climate proxies that suggest, around 430 ky, a glacial cold period that may have been more severe than the LGM [44,45]. Coalescence of regional clades around 430 ky was also observed in *Maoricicada campbelli*[29]. This hypothesis could be tested more effectively by multi-gene datasets allowing more sensitive estimates of divergence times.

Dispersal ability may also have influenced population genetic structure in the *AmphipsaltaNotopsalta* species, contributing to lower genetic differentiation in the forest taxa *A. zelandica* and *A. cingulata*. Lineage-specific differences in traits affecting dispersal and gene flow should influence relative rates of allopatric divergence, especially in neutral genetic characters [46,47]. The two forest taxa are the largest-bodied cicadas in New Zealand (see photo inset in Figure 5), and they are likely the strongest fliers, suggesting that greater dispersal ability leads to greater gene flow among populations and reduced rates of allopatric differentiation in these species. However, the mtDNA data show that dispersal in *A. cingulata* and *A. zelandica* has not completely prevented the formation of intraspecific phylogeographic structure (Table 2, Figure 3 and Figure 5). All cicadas spend multiple years as juveniles underground, a life-history feature that must reduce population vagility compared to similarly sized insects [48].

#### Mid-Pleistocene diversification of open-habitat species

In *Notopsalta sericea* and *Amphipsalta strepitans*, more intraspecific lineages are evident than in the two forest taxa, and multiple lineages apparently persisted through the hypothesized Pleistocene minimum of 430 ky. In both cases, the credible intervals on mtDNA coalescence include the period from about 0.6-2.0 Ma (mean 1.4 and 1.2 Ma, respectively) (Table 3).

Multiple geological and climatic changes are known to have occurred around 1 Ma that could have affected diversification of these open-habitat species [49]. Pleistocene climates became progressively colder and drier, suggesting the continued replacement of forests by shrub and grassland habitats. Large sections of NI and northern SI began a period of accelerated uplift that has continued to the present [49,50], increasing rain shadow effects across eastern provinces and expanding the dry slope habitats used by *A. strepitans* and *N. sericea*. The puzzling mtDNA homogeneity of *N. sericea* populations across the northern third of NI, a reversal of the “northern richness, southern purity” pattern frequently observed in NZ [51-55], could be explained by very recent acceleration of uplift in that area [56] leading to northward dispersal from source populations in central NI.

Unlike *A. zelandica* and other forest insects whose LGM distributions have been modeled [39,40,52,55], for *A. strepitans* the hypothesized LGM range was not smaller than the present-day range (Figure 6). A higher degree of phylogeographic structuring relative to the other cicadas was also detected, consistent with survival of multiple distinct populations over a longer period, as has been detected in other non-forest invertebrates (e.g., [46,57-59]). *A. strepitans* was the only cicada for which temperature explained less than 15% of model fit, suggesting its wider LGM distribution may have been related to success in cooler temperatures, although tolerance of aridity could also be involved. Greater success in drier climates was offered to explain a larger LGM distribution implied by microsatellite data for the NZ tree species *Pseudopanax ferox*[60]. The ENM failed to capture the southern extent of *A. strepitans’* current distribution, possibly because of the low number of localities from South Canterbury and Otago relative to the well-sampled northeastern SI, limiting the inferences we can make about the LGM projection. However, the low sample numbers in the south likely indicate genuine scarcity in this region, and the discovery of additional southern localities would likely expand, rather than contract, the projected climatic distribution of this species.

### Ecological niche models and LGM refugia

No LGM refugia were projected for *N. sericea* or *A. cingulata*, despite apparently successful model performance, implying that no counterpart of either species’ current realized niche existed during the LGM. Yet genetic patterns suggest that both species survived on NI during that period, and *N. sericea* contains considerable mtDNA phylogeographic structure on NI. Three hypotheses (which are not mutually exclusive) can explain these surprising results: 1) the species have only recently occupied their current climatic niche (niche evolution), 2) they survived in isolated microrefugia undetectable using these methods, or 3) their current realized niches inadequately represent the fundamental niche of each species (environmental disequilibrium). The first hypothesis is less likely; existing evidence for insects, while largely based on beetles, offers little evidence of post-LGM shifts in climate exploitation [61,62].

The second and third hypotheses are both plausible, but difficult to disentangle. *Amphipsalta cingulata* and *Notopsalta sericea* survived somewhere in NZ, and it is likely that local habitat attributes created small refugia undetectable using our elevation-scaled meteorological data [63]. Past climates may have had different combinations of environmental variables than exist today, yielding predictions of past ranges that are under- or overestimates [31,64]. In addition, ENMs as applied here are a representation of each species’ climatic niche, but if a species is restricted from exploiting its full climatic niche due to other factors, projections onto past climates may exclude potentially habitable areas [64]. Several cicada species are present in the southernmost NI but are absent from SI (see maps in [1]), indicating that Cook Strait is an important dispersal barrier even for flighted species [31], and the absence of *A. cingulata* and *N. sericea* from cooler and wetter conditions in SI would result in underrepresentation of suitable LGM refugia. Excluding these potentially habitable, unoccupied regions from the training data reduces the risk of over-fitting but increases model uncertainty (clamping; [65]) and thus, transferability of the model [66], and for both species, restricting the training data resulted in clamping (extensively so for *N. sericea*) where none was previously detected. Interestingly, neither species has extended its range into the cooler mid- or high elevations of NI, and restricting the training data failed to yield additional refugia, suggesting that spurious over-fitting of the climate data was not the key driver of this absence, although it cannot be conclusively ruled out. Projections of ENMs onto past climates must be interpreted with caution, particularly where their congruence with external data (e.g., DNA) is low.

## Conclusions

This study and others (e.g., [41]) show that closely related and regionally co-occurring organisms can experience dramatically different diversification rates over a substantial period (here, mid-Miocene to present), a conclusion presaged by earlier literature on species distributions (e.g., [67]). Because the two NZ cicada lineages colonized the landscape independently, the difference in the number of extant species is not simply a side-effect of the geography of initial diversification, as is possible with sister-clades [68]. Apparently, many physiological, ecological, and historical factors interact to influence diversification. In the case of the two NZ cicada radiations, we speculate that differences in climatic tolerances/preferences of the two ancestors could have persistently influenced the geographic ranges of the two radiating lineages. Climatic refugia were not detected for two of the four species, suggesting that Pleistocene survival and lineage diversification were moderated by factors other than broad regional climatic conditions. This study also adds to a growing number of phylogeographic analyses implicating mountain-building and Pleistocene habitat fragmentation as drivers of diversification in NZ insects.

## Methods

### Sample collection and range estimation

*Amphipsalta* and *Notopsalta* specimens (typically male) were collected during surveys from 1993–2010. The species *Kobonga apicata* (Ashton) (see [69]), from central and western Australia, was selected as an outgroup based on phylogenetic analyses at the tribe level (unpublished data). Tissue collections were sought from sites distributed as uniformly as possible within each species' range. When a species was detected but not collected, the presence of its diagnostic song was noted and digitally recorded if possible. Localities used for ecological niche modeling were based on collection and song data from this study and historical records [11] (Figure 2; see Additional file 2: Appendix for details).

### Genetic sequencing

Genomic DNA was extracted from 1–2 legs using the Qiagen DNeasy Tissue Kit and protocol (Qiagen, Valencia, California, USA), except that the digestion was conducted over 3–18 hours at 54 °C. Approximately 1350 base pairs of the mitochondrial cytochrome oxidase I (COI) gene were amplified in one of two ways: (1) in two sections; the 5’ (barcoding) section with primers C1-J-1490 + C1-N-2198 [70], annealing temperature 50°C, and the 3’ section with primers C1-J-2195 + TL2-N-3014 [71], annealing temperature 56°C; (2) in one piece using C1-J-1490 + TL2-N-3014, annealing temperature 45°C. Amplified products were cleaned using the Clontech Extract II Kit (Clontech, Mountain View, CA, USA) or ExoSAP-IT (USB Corp., Cleveland, OH). For examination of the species-level *AmphipsaltaNotopsalta* relationships, a subset (see Additional file 2: Appendix) of the individuals sampled were sequenced for two nuclear genes, Calmodulin (763 bp) and elongation factor-1 alpha (EF-1α; ca. 1400 bp). Calmodulin was amplified using the primers Cal60For [16] and Cal2Rev (UBC insect primer kit). A touchdown cycle was used with the following parameters: (1) a 94°C hold for 2 min.; (2) a touchdown PCR sequence of 94°C for 45 sec., 60-50°C for 45 sec., and 72°C for 1 min. – decreasing by 1°C every cycle and repeated for 10 cycles; (3) a steady PCR sequence of 94°C for 45 sec., 50°C for 45 sec., and 72°C for 1 min., repeated for 25 cycles; and (4) a 72°C hold for 10 min. For *A. cingulata*, internal calmodulin primers were created from the sequence of the other cicadas. EF-1α was amplified in two overlapping sections using the primers EF1-F001-cicada + EF1-R752 [5], annealing temperature 53°C, and EF1-PA-f650ambig [72] + EF1-N-1419 [73], annealing temperature 58°C. Cycle sequencing was conducted using the Applied Biosystems Big Dye Terminator v1.1 cycle sequencing kit at 1/8- to 1/4-scale reaction volume, and the product was cleaned by Sephadex filtration (GE Healthcare, Piscataway, NJ, USA) and visualized on an Applied Biosystems ABI 3100 capillary sequencer. Sequences were analyzed using ABI Prism Sequencing Analysis software v3.7 (Applied Biosystems), edited in Sequencher v3.1 (Gene Codes Corp., Ann Arbor, MI), aligned in MacClade [74] and checked by eye.

### Phylogenetic estimation

Individual gene-trees were estimated using partitioned maximum-likelihood (ML) in Garli version 2.0 [75], using datasets that contained all unique haplotypes. Each nuclear gene was modeled using a single partition because of the low number of parsimony-informative sites available for estimating parameters (see Results); substitution models for these genes were selected using Modeltest v3.7 [76]. For the mtDNA data, alternative combinations of partition schemes and partition-specific substitution models, based on the three codon-position categories, were tested using the “greedy” algorithm of the program PartitionFinder v1.0 [77] and the BIC criterion, with the branch lengths of alternative partitions linked and with the software set to evaluate the full substitution model set. Default settings were used for Garli analyses, except that the number of generations required for termination (*genthreshfortopoterm*) was increased to 40,000 to improve the search. ML analyses in Garli offered estimates of branch lengths that were not influenced by Bayesian priors [78,79]. The number of discrete gamma categories was set to 8 to improve resolution of site rates over the default setting of 4 categories. For each analysis, 100 bootstrap pseudoreplicates were conducted in Garli, and these were summarized on the ML tree with the program SumTrees v3.3.1, part of the DendroPy package [80].

### Pairwise genetic divergence plots

Because “saturation” of genetic data by multiple hits has been observed to influence divergence time estimates [81,82], we constructed plots of uncorrected genetic distances calculated in Mesquite v. 2.75 [83] vs. ML model-corrected patristic (tree-based) distances for the *AmphipsaltaNotopsalta* COI dataset and a published, combined COI + COII ingroup dataset of about the same size (bp) from *Kikihia*[1] containing 30 species. Maximum uncorrected genetic distances from the *Kikihia* dataset are similar to those in *AmphipsaltaNotopsalta*.

### Divergence-time estimation

To place the *Amphipsalta-Notopsalta* tree in an approximate time frame, Bayesian relaxed-clock phylogenetic analyses of the full COI dataset, with identical haplotypes and the outgroup included, were conducted using BEAST v1.6.1 [84]. Because no fossil or geological calibrations are available for dating these cicadas, the analysis was calibrated using COI molecular clock estimates from the literature. While many insect studies have used the 0.0115 substitutions/site/my/lineage clock rate estimated by Brower [17], more recent studies have suggested both lower (e.g., [19,85]) and higher rates [25] (Table 5), in part because of the use of highly partitioned sequence-evolution models [25] that tend to reconstruct more change along branches. In each analysis, the mean and standard deviation of the relaxed clock were fixed to reflect the literature estimates from Table 5 by the following settings: (1) we set *ucld.mean* (the mean of the lognormal prior distribution on branch rates) to 0.0115 substitutions/site/my, the Brower [17] rate, (2) we set *ucld.stdev*, the standard deviation of the lognormal prior on branch rates, to 0.3, so that the 95% confidence interval of the lognormal distribution included rates from 0.006 to 0.0188 substitutions/site/my, and (3) we deactivated the operators for *ucld.mean* and *ucld.stdev*, so that the software did not attempt to estimate these parameters. We suggest that the approach of combining all empirical knowledge into a single prior is superior to the use of separate analyses under alternative rates an approach that was used in earlier work on *Kikihia*[1].

BEAST analyses of the COI dataset used the same three-partition scheme as the ML analysis. The *Amphipsalta* sequences were constrained to form a monophyletic clade, as supported by the ML analysis (see Results), because initial runs of some models suggested a tendency for BEAST to place the ingroup root between clades containing *A. cingulata* + *A. zelandica* and *N. sericea* + *A. strepitans*. Model parameters and partition rate multipliers were estimated separately for each partition. Four tree priors were tested in separate analyses and compared using the Bayes factor scores calculated in Tracer v1.5 [86]: Yule, birth-death, a constant-size coalescent, and an expansion-growth coalescent. The criterion 2 ln Bayes factor > 10 [87] was used to decide if a given model was superior. Models were run to 40 million generations, and stationarity and adequate sample sizes (i.e., > 200) were confirmed using Tracer [86] and by ensuring that multiple runs found the same solution. Other priors were left at the default values specified by BEAUti v 1.6.1 (provided with the BEAST package). Statistically improper priors, such as intervals extending to infinity, were converted to proper priors with a large, finite upper bound. Prior distributions were estimated by running the analysis with no data.

### Test for intraspecific diversification

Because phylogeographic patterns in the mtDNA dataset suggested ongoing intraspecific allopatric diversification, we tested for the threshold between interspecific and intraspecific lineage dynamics using the generalized mixed Yule-coalescent (GMYC) model of Pons et al. [32] as implemented in the R package SPLITS [88,89]. If the samples of each species are drawn from a single panmictic population, the method should identify a branching-rate shift at the base of each species caused by the change from coalescent (intraspecific) to birth-death (interspecific) dynamics. A tree containing only unique haplotypes was analyzed in BEAST to obtain a chronogram under the same model used in the divergence-time analysis above. The chronogram was tested in the GMYC analysis using an interval of 0,10 and a single-threshold model.

Geographic structure in the mtDNA dataset was also tested using an analysis of molecular variance (AMOVA) as implemented in Arlequin v 3.1 [90]. Populations were combined into groups according to a six-region scheme defined by Cook Strait and latitude lines -37° S, -39° S, -43° S, and -45° S.

### Ecological niche modeling

Combining ecological niche models with paleoclimatic data to hindcast species ranges offers a means of testing refugia hypotheses developed from genetic information. For comparison with intraspecific phylogeographic patterns, the current and LGM distributions of each species were estimated using ecological niche models. Climate data used to construct the ENMs included mean annual rainfall (mm), mean February rainfall (mm), mean annual solar radiation (kJ/day/m^2^), mean annual temperature (°C), mean February temperature (°C), minimum temperature of the coldest month (°C, July), and October vapor pressure deficit (kPa), all at 100 m resolution [91]. These layers, derived from meteorological data (1950–1980, [91]), were fitted to the LGM based on estimates of temperature depression from marine isotope stages and estimates of LGM topography based on depression of the sea level by 120 m (J.R. Leathwick, unpublished data, see [39]).

Ecological niche models were generated with Maxent 3.3.1 [92,93]. Models were built using 341 *A. zelandica*, 62 *A. strepitans*, 86 *A. cingulata* and 87 *Notopsalta sericea* localities. Maximum iterations were increased as necessary to allow the algorithm to converge, with default settings used otherwise. To ensure consistency of model predictions among repeated runs, we performed a 10-fold cross-validation, in which a different 90% of localities were used to train the model and 10% were used to test it for each of 10 runs, such that each locality was used to test the model once. Estimated models for each species were projected onto LGM climate surfaces for all 10 runs, using the "fade by clamping" setting for output grids, to visually inspect for differences in regions projected as suitable habitat between replicate runs. "Clamping" refers to the handling of LGM grid sections with climatic values outside the range observed during training – Maxent sets these to the corresponding maximum or minimum observed training value. The "fade by clamping" method attempts to correct for the effect of this uncertainty on the projected distribution by reducing the estimated climatic suitability proportional to the level of clamping. Clamping grids were also inspected for differences in model output under different data partitions. The final geographical projections represent the mean point-wise prediction over ten model runs. Model performance was evaluated using threshold-dependent binomial omission tests and the Area Under the (Receiver Operating Characteristic) Curve (AUC) calculated by Maxent.

Regions which contain suitable climate conditions for a species may be unoccupied due to other factors, such as dispersal barriers [94]. Inclusion of these regions during model calibration can result in overfitting to conditions found near the collection localities [65]. When hindcasting to LGM climate conditions indicated no refugia for the two taxa limited to NI (*A. cingulata* and *N. sericea*; see below), we re-ran these analyses using NI climate data only for training and testing to examine whether the model for these species was being overfitted by the inclusion of SI data. The model was then projected onto LGM surfaces for all of NZ. While Cook Strait is not likely to be an absolute dispersal barrier to cicadas, it could delay colonization of SI by species restricted to northern NI refugia.

## Competing interests

All authors declare that no competing financial or non-financial interests exist.

## Authors' contributions

DM collected specimens, conceived the study, conducted phylogenetic and phylogeographic analyses, and drafted the manuscript. KH collected specimens, conceived the study, conducted genetic sequencing, and drafted the manuscript. KM conceived the study, conducted the ENM analyses and drafted the manuscript. CC conducted genetic sequencing and assisted with phylogenetic and phylogeographic analyses. TB collected specimens, assisted with the analyses, and drafted the manuscript. CS collected specimens, conceived and coordinated the study and its funding, assisted with the analyses, and drafted the manuscript. All authors read and approved the final manuscript.

## Supplementary Material

Additional file 1 **Figure S1.** Population-lineages diagnosed within the four described species using the generalized mixed Yule-coalescent algorithm. Connected red branches indicate haplotypes contained within a single diagnosed population. Results varied considerably depending on the particular tree used (e.g., all sequences, haplotypes only, *A. strepitans*-*N. sericea* only) but multiple lineages were always detected within the four described species. Click here for file

Additional file 2 ** Appendix Specimen locality data.** Asterisked latitude indicates that GPS was estimated from a printed map. Elevations were recorded in meters. Click here for file
