# Supplementary material for: Limited, episodic diversification and contrasting phylogeography in a New Zealand cicada radiation
Source: BMC Evol Biol. 2012 Sep 11;12:177. doi: 10.1186/1471-2148-12-177 (PMC3537654; doi:10.1186/1471-2148-12-177)
Supplement: Additional file 2 — Appendix Specimen locality data. Asterisked latitude indicates that GPS was estimated from a printed map. Elevations were recorded in meters. [file 1471-2148-12-177-S2.doc]

Online Appendix 1. Specimen locality data. Asterisked latitude indicates that GPS was estimated from a printed map. Elevations were recorded in meters.

| GENUS | SPECIES | SPECIMEN CODE | LATITUDE | LONGITUDE | DISTRICT | LOCATION | ELEVATION | DATE |
| --- | --- | --- | --- | --- | --- | --- | --- | --- |
| *Amphipsalta* | *cingulata* | 01.NZ.ND.RUS.01 | -35.257 | 174.121666 | Northland | Russell | N/A | 2/24/01 |
| *Amphipsalta* | *cingulata* | 01.NZ.ND.RUS.02 | -35.257 | 174.121666 | Northland | Russell | N/A | 2/24/01 |
| *Amphipsalta* | *cingulata* | 01.NZ.WN.NEV.05 | -41.302 | 174.829216 | Wellington | Wellington | 100 | 2/1/01 |
| *Amphipsalta* | *cingulata* | 03.NZ.ND.DAR.03 | -35.954 | 173.849033 | Northland | near Dargaville | 46 | 3/19/03 |
| *Amphipsalta* | *cingulata* | 03.NZ.ND.TPT.03 | -34.439 | 172.713633 | Northland | Tapotupotu Bay | 22 | 3/20/03 |
| *Amphipsalta* | *cingulata* | 05.NZ.AK.FMR.01 | -37.239 | 175.38575 | Auckland | Waitakaruru | 24 | 3/19/05 |
| *Amphipsalta* | *cingulata* | 05.NZ.AK.HER.01 | -37.842 | 174.733333 | Auckland | Herne Bay, Auckland | N/A | 2/20/05 |
| *Amphipsalta* | *cingulata* | 05.NZ.AK.LAN.01 | -36.883 | 174.848283 | Auckland | Glen Innes, Auckland | 53 | 3/21/05 |
| *Amphipsalta* | *cingulata* | 05.NZ.AK.OKW.01 | -36.346 | 174.281883 | Auckland | SW of Wellsford | 7 | 12/18/05 |
| *Amphipsalta* | *cingulata* | 05.NZ.BP.ERI.01 | -38.044 | 176.486983 | Bay of Plenty | Lake Rotoiti | 296 | 3/6/05 |
| *Amphipsalta* | *cingulata* | 05.NZ.CL.SBR.03 | -36.510 | 175.440883 | Coromandel | Sandy Bay, nr. Port Charles | 166 | 3/20/05 |
| *Amphipsalta* | *cingulata* | 05.NZ.CL.SBR.05 | -36.510 | 175.440883 | Coromandel | Sandy Bay, nr. Port Charles | 166 | 3/20/05 |
| *Amphipsalta* | *cingulata* | 05.NZ.GB.GBB.01 | -38.673 | 178.00315 | Gisborne | Midway Beach, Gisborne | 6 | 3/9/05 |
| *Amphipsalta* | *cingulata* | 05.NZ.GB.GBB.02 | -38.673 | 178.00315 | Gisborne | Midway Beach, Gisborne | 6 | 3/9/05 |
| *Amphipsalta* | *cingulata* | 05.NZ.HB.JON.01 | -39.542 | 176.863616 | Hawke’s Bay | Meeanee, nr. Napier | 11 | 3/10/05 |
| *Amphipsalta* | *cingulata* | 05.NZ.HB.KRK.03 | -39.943 | 176.930216 | Hawke’s Bay | Kairakau Beach | 5 | 3/10/05 |
| *Amphipsalta* | *cingulata* | 05.NZ.HB.KRK.04 | -39.943 | 176.930216 | Hawke’s Bay | Kairakau Beach | 5 | 3/10/05 |
| *Amphipsalta* | *cingulata* | 05.NZ.ND.DBR.03 | -34.956 | 173.374383 | Northland | Rd. to Karikari Peninsula | 29 | 3/22/05 |
| *Amphipsalta* | *cingulata* | 05.NZ.TK.RWY.11 | -39.058 | 174.057966 | Taranaki | New Plymouth | 20 | 3/18/05 |
| *Amphipsalta* | *cingulata* | 05.NZ.TO.WFD.04 | -38.287 | 175.674916 | Taupo | Waipapa Falls power station | 189 | 12/12/05 |
| *Amphipsalta* | *cingulata* | 05.NZ.WN.WIB.01 | -40.861 | 175.02335 | Wellington | Waikanae Beach | 3 | 3/14/05 |
| *Amphipsalta* | *cingulata* | 05.NZ.WO.MKP.03 | -38.306 | 174.719133 | Waikato | Waikanae Beach | 15 | 3/18/05 |
| *Amphipsalta* | *cingulata* | 06.NZ.ND.KER.02 | -35.235 | 173.95555 | Northland | Kerikeri | 47 | 12/14/06 |
| *Amphipsalta* | *cingulata* | 07.NZ.TK.RWY.05 | -39.058 | 174.057966 | Taranaki | New Plymouth | 20 | 3/1/07 |
| *Amphipsalta* | *cingulata* | 97.NZ.TO.WPE.53 | -38.890 | 175.9545 | Taupo | Moturere reserve | 430 | 1/26/97 |
| *Amphipsalta* | *strepitans* | 00.NZ.MC.CAP.58 | -43.788 | 172.875 | Mid Canterbury | nr. Akaroa | N/A | 1/19/00 |
| *Amphipsalta* | *strepitans* | 02.NZ.KA.CON.01 | -42.600 | 173.417733 | Kaikoura | Conway River, SH1 | 56 | 12/19/02 |
| *Amphipsalta* | *strepitans* | 04.NZ.KA.CLA.05 | -42.142 | 173.8799 | Kaikoura | W. of Clarence | 45 | 2/19/04 |
| *Amphipsalta* | *strepitans* | 04.NZ.MB.ROS.01 | -41.681 | 173.960166 | Marlborough | Awatere Vly | 121 | 2/14/04 |
| *Amphipsalta* | *strepitans* | 04.NZ.MB.ROS.03 | -41.681 | 173.960166 | Marlborough | Awatere Vly | 121 | 2/14/04 |
| *Amphipsalta* | *strepitans* | 04.NZ.MB.WEL.03 | -41.777 | 173.833383 | Marlborough | Awatere Vly | 243 | 2/14/04 |
| *Amphipsalta* | *strepitans* | 06.NZ.MB.PVE.01 | -41.522 | 173.611633 | Marlborough | Wairau Vly | 112 | 1/3/06 |
| *Amphipsalta* | *strepitans* | 06.NZ.MB.PVE.02 | -41.522 | 173.611633 | Marlborough | Wairau Vly | 112 | 1/3/06 |
| *Amphipsalta* | *strepitans* | 05.NZ.MC.RHW.03 | -43.519 | 171.646433 | Mid Canterbury | S of Rakaia Gorge | 364 | 2/4/05 |
| *Amphipsalta* | *strepitans* | 05.NZ.NC.JAC.03 | -42.857 | 172.517666 | North Canterbury | E of Jacks Saddle | 370 | 2/13/05 |
| *Amphipsalta* | *strepitans* | 05.NZ.NC.JAC.04 | -42.857 | 172.517666 | North Canterbury | E of Jacks Saddle | 370 | 2/13/05 |
| *Amphipsalta* | *strepitans* | 06.NZ.KA.WMA.12 | -41.883 | 174.045266 | Kaikoura | W of Te Rapa, E of Kilgram | 65 | 1/5/06 |
| *Amphipsalta* | *strepitans* | 06.NZ.KA.WMA.17 | -41.883 | 174.045266 | Kaikoura | W of Te Rapa, E of Kilgram | 65 | 1/5/06 |
| *Amphipsalta* | *strepitans* | 06.NZ.NC.LEW.01 | -43.242 | 172.204016 | North Canterbury | SW Lees Valley | 374 | 1/7/06 |
| *Amphipsalta* | *strepitans* | 07.NZ.CO.ROX.03 | -45.446 | 169.307133 | Central Otago | N. of Roxburgh | 272 | 2/13/07 |
| *Amphipsalta* | *strepitans* | 07.NZ.NC.SWH.01 | -42.576 | 172.719233 | North Canterbury | SW of Hanmer Springs | 328 | 2/15/07 |
| *Amphipsalta* | *strepitans* | 09.NZ.DN.PUK.01 | -45.380 | 170.11 | Dunedin | SE of Pukerangi | N/A | 12/6/09 |
| *Amphipsalta* | *strepitans* | 09.NZ.DN.PUK.02 | -45.380 | 170.11 | Dunedin | SE of Pukerangi | N/A | 12/6/09 |
| *Amphipsalta* | *strepitans* | 93.NZ.WN.ORO.07 | -41.412 | 174.904166 | Wellington | Orongorongo River mouth | 10 | 1/12/93 |
| *Amphipsalta* | *strepitans* | 93.NZ.WN.ORO.08 | -41.412 | 174.904166 | Wellington | Orongorongo River mouth | 10 | 1/12/93 |
| *Amphipsalta* | *strepitans* | 94.NZ.WN.RED.01 | -41.358 | 174.725 | Wellington | NE of Sinclair Head | N/A | 2/5/94 |
| *Amphipsalta* | *strepitans* | 94.NZ.WN.RED.02 | -41.358 | 174.725 | Wellington | NE of Sinclair Head | N/A | 2/5/94 |
| *Amphipsalta* | *strepitans* | 94.NZ.WN.RED.03 | -41.358 | 174.725 | Wellington | NE of Sinclair Head | N/A | 2/5/94 |
| *Amphipsalta* | *strepitans* | 94.NZ.WN.RED.04 | -41.358 | 174.725 | Wellington | NE of Sinclair Head | N/A | 2/5/94 |
| *Amphipsalta* | *zelandica* | 01.NZ.KA.BDS.14 | -42.275 | 173.771183 | Kaikoura | Blue Duck Reserve | 63 | 2/7/01 |
| *Amphipsalta* | *zelandica* | 01.NZ.WN.RIM.02 | -41.115 | 175.232066 | Wellington | Rimutaka Summit Trail | 561 | 1/30/01 |
| *Amphipsalta* | *zelandica* | 02.NZ.NN.UCA.01 | -40.694 | 172.379166 | Nelson | S. of Paturau River (town) | 72 | 2/4/02 |
| *Amphipsalta* | *zelandica* | 02.NZ.NN.UCA.02 | -40.694 | 172.379166 | Nelson | S. of Paturau River (town) | 72 | 2/4/02 |
| *Amphipsalta* | *zelandica* | 02.NZ.NN.UCA.03 | -40.694 | 172.379166 | Nelson | S. of Paturau River (town) | 72 | 2/4/02 |
| *Amphipsalta* | *zelandica* | 02.NZ.NN.UCA.04 | -40.694 | 172.379166 | Nelson | S. of Paturau River (town) | 72 | 2/4/02 |
| *Amphipsalta* | *zelandica* | 02.NZ.SD.CUL.20 | -41.274 | 173.787866 | Marlborough Sounds | E. of Havelock | 178 | 2/6/02 |
| *Amphipsalta* | *zelandica* | 02.NZ.SD.CUL.21 | -41.274 | 173.787866 | Marlborough Sounds | E. of Havelock | 178 | 2/6/02 |
| *Amphipsalta* | *zelandica* | 02.NZ.SD.CUL.22 | -41.274 | 173.787866 | Marlborough Sounds | E. of Havelock | 178 | 2/6/02 |
| *Amphipsalta* | *zelandica* | 02.NZ.SD.CUL.23 | -41.274 | 173.787866 | Marlborough Sounds | E. of Havelock | 178 | 2/6/02 |
| *Amphipsalta* | *zelandica* | 02.NZ.SD.CUL.24 | -41.274 | 173.787866 | Marlborough Sounds | E. of Havelock | 178 | 2/6/02 |
| *Amphipsalta* | *zelandica* | 02.NZ.WD.KEL.05 | -42.805 | 171.5741 | Westland | N. of Otira | 350 | 2/14/02 |
| *Amphipsalta* | *zelandica* | 03.NZ.KA.KVR.02 | -41.972 | 173.9862 | Kaikoura | NW of Kekerengu | 43 | 2/18/03 |
| *Amphipsalta* | *zelandica* | 03.NZ.ND.WAI.01 | -35.655 | 173.570183 | Northland | Waipoua Forest | 102 | 3/19/03 |
| *Amphipsalta* | *zelandica* | 04.NZ.BR.PRC.01 | -42.109 | 171.3361 | Buller | Punakaiki | 14 | 2/16/04 |
| *Amphipsalta* | *zelandica* | 04.NZ.KA.CWF.01 | -42.658 | 173.443433 | Kaikoura | 37 km S. of Kaikoura | 28 | 2/18/04 |
| *Amphipsalta* | *zelandica* | 04.NZ.MC.AAB.03 | -43.716 | 172.745116 | Mid Canterbury | Banks Peninsula | 320 | 2/12/04 |
| *Amphipsalta* | *zelandica* | 04.NZ.MC.AAC.02 | -43.707 | 172.750916 | Mid Canterbury | Banks Peninsula | 750 | 2/12/04 |
| *Amphipsalta* | *zelandica* | 04.NZ.MC.AKR.02 | -43.804 | 172.969216 | Mid Canterbury | Akaroa | 14 | 2/21/04 |
| *Amphipsalta* | *zelandica* | 04.NZ.NN.GLF.01 | -41.289 | 173.328983 | Nelson | E. of Nelson | 35 | 2/20/04 |
| *Amphipsalta* | *zelandica* | 05.NZ.BP.MTP.03 | -38.093 | 177.486183 | Bay of Plenty | SE of Opotiki | 443 | 3/7/05 |
| *Amphipsalta* | *zelandica* | 05.NZ.BP.MTP.04 | -38.093 | 177.486183 | Bay of Plenty | SE of Opotiki | 443 | 3/7/05 |
| *Amphipsalta* | *zelandica* | 05.NZ.CL.SBR.01 | -36.510 | 175.440883 | Coromandel | NW of Port Charles | 166 | 3/20/05 |
| *Amphipsalta* | *zelandica* | 05.NZ.CL.SBR.04 | -36.510 | 175.440883 | Coromandel | NW of Port Charles | 166 | 3/20/05 |
| *Amphipsalta* | *zelandica* | 05.NZ.GB.TRZ.01 | -37.866 | 178.1986 | Gisborne | NW of Ruatoria | 123 | 3/8/05 |
| *Amphipsalta* | *zelandica* | 05.NZ.GB.TRZ.02 | -37.866 | 178.1986 | Gisborne | NW of Ruatoria | 123 | 3/8/05 |
| *Amphipsalta* | *zelandica* | 05.NZ.GB.WGH.16 | -38.733 | 177.827266 | Gisborne | SW of Gisborne | 29 | 3/9/05 |
| *Amphipsalta* | *zelandica* | 05.NZ.HB.MAN.01 | -40.001 | 176.873416 | Hawke’s Bay | S. of Hastings | 45 | 3/10/05 |
| *Amphipsalta* | *zelandica* | 05.NZ.NN.PIG.01 | -40.825* | 172.966666 | Nelson | Abel Tasman NP | N/A | 2/20/05 |
| *Amphipsalta* | *zelandica* | 05.NZ.NN.PIG.02 | -40.825* | 172.966666 | Nelson | Abel Tasman NP | N/A | 2/20/05 |
| *Amphipsalta* | *zelandica* | 05.NZ.NN.PIG.03 | -40.825* | 172.966666 | Nelson | Abel Tasman NP | N/A | 2/20/05 |
| *Amphipsalta* | *zelandica* | 05.NZ.SD.NYD.01 | -41.233 | 173.766666 | Marlborough Sounds | N of Havelock | N/A | 1/29/05 |
| *Amphipsalta* | *zelandica* | 05.NZ.SD.NYD.02 | -41.233 | 173.766666 | Marlborough Sounds | N of Havelock | N/A | 1/29/05 |
| *Amphipsalta* | *zelandica* | 05.NZ.TK.PAP.01 | -38.964 | 174.926816 | Taranaki | E of Paparata | 305 | 3/5/05 |
| *Amphipsalta* | *zelandica* | 05.NZ.TK.SME.01 | -39.240 | 174.5861 | Taranaki | E of Strathmore | 175 | 3/4/05 |
| *Amphipsalta* | *zelandica* | 05.NZ.WA.HPW.01 | -41.434 | 175.226183 | Wairarapa | 4km N of Te Humenga Pt | 124 | 3/12/05 |
| *Amphipsalta* | *zelandica* | 05.NZ.WD.BFN.01 | -44.008333* | 169.391666 | Westland | SH6, N of Haast Pass | N/A | 1/15/05 |
| *Amphipsalta* | *zelandica* | 05.NZ.WN.EMA.05 | -40.575 | 175.490466 | Wellington | nr. Tokomaru Reservoir Dam | 340 | 3/15/05 |
| *Amphipsalta* | *zelandica* | 05.NZ.WN.EMA.07 | -40.575 | 175.490466 | Wellington | nr. Tokomaru Reservoir Dam | 340 | 3/15/05 |
| *Amphipsalta* | *zelandica* | 05.NZ.WO.MNW.01 | -38* | 175.083333 | Waikato | Mt Pirongia | N/A | 2/28/05 |
| *Amphipsalta* | *zelandica* | 06.NZ.CL.LBV.01 | -36.217 | 175.058333 | Coromandel | Little Barrier Island | N/A | 11/27/06 |
| *Amphipsalta* | *zelandica* | 06.NZ.NC.NLC.01 | -42.544 | 173.452216 | North Canterbury | 25km S of Kaikoura | N/A | 2/12/06 |
| *Amphipsalta* | *zelandica* | 06.NZ.ND.HEF.02 | -35.16305* | 173.26945 | Northland | Mangataiore, ESE of Kaitaia | N/A | 2/7/06 |
| *Amphipsalta* | *zelandica* | 06.NZ.ND.HEF.03 | -35.16305* | 173.26945 | Northland | Mangataiore, ESE of Kaitaia | N/A | 2/7/06 |
| *Amphipsalta* | *zelandica* | 06.NZ.ND.HEF.04 | -35.16305* | 173.26945 | Northland | Mangataiore, ESE of Kaitaia | N/A | 2/7/06 |
| *Amphipsalta* | *zelandica* | 06.NZ.ND.HEF.05 | -35.16305* | 173.26945 | Northland | Mangataiore, ESE of Kaitaia | N/A | 2/7/06 |
| *Amphipsalta* | *zelandica* | 06.NZ.ND.KEE.01 | -35.222 | 174.049416 | Northland | N of Kerikeri Inlet (town) | 45 | 12/14/06 |
| *Amphipsalta* | *zelandica* | 06.NZ.ND.MGO.01 | -35.189466* | 173.455866 | Northland | Mangamuka Gorge Res. | N/A | 2/6/06 |
| *Amphipsalta* | *zelandica* | 06.NZ.SL.TUA.01 | -46.109416* | 167.691066 | Southland | Tuatapere Reserve, SH96 | N/A | 1/18/06 |
| *Amphipsalta* | *zelandica* | 06.NZ.WD.DMC.01 | -43.577 | 169.802233 | Westland | SW of Fox Glacier | 55 | 1/10/06 |
| *Amphipsalta* | *zelandica* | 06.NZ.WD.MCP.01 | -43.956083* | 169.313916 | Westland | NNW of Haast Pass | N/A | 1/13/06 |
| *Amphipsalta* | *zelandica* | 06.NZ.WD.PLF.01 | -44.01225* | 169.381716 | Westland | Pleasant Flat, SH6 | N/A | 1/13/06 |
| *Amphipsalta* | *zelandica* | 07.NZ.BR.INA.01 | -42.271 | 171.9672 | Buller | SE of Reefton | 383 | 2/16/07 |
| *Amphipsalta* | *zelandica* | 07.NZ.BR.INJ.01 | -41.860 | 171.974416 | Buller | E of Inangahua Jct, SH6 | 90 | 2/16/07 |
| *Amphipsalta* | *zelandica* | 07.NZ.BR.OGR.05 | -42.078 | 171.824966 | Buller | N of Reefton | 160 | 2/16/07 |
| *Amphipsalta* | *zelandica* | 07.NZ.BR.RSE.02 | -42.147 | 171.898716 | Buller | SE of Reefton | 222 | 2/16/07 |
| *Amphipsalta* | *zelandica* | 07.NZ.DN.WPA.02 | -45.930 | 170.055433 | Dunedin | NW of Lake Waipori | 39 | 2/19/07 |
| *Amphipsalta* | *zelandica* | 07.NZ.DN.WPA.03 | -45.930 | 170.055433 | Dunedin | NW of Lake Waipori | 39 | 2/19/07 |
| *Amphipsalta* | *zelandica* | 07.NZ.DN.WPA.04 | -45.930 | 170.055433 | Dunedin | NW of Lake Waipori | 39 | 2/19/07 |
| *Amphipsalta* | *zelandica* | 07.NZ.GB.MAO.02 | -38.784 | 177.1344 | Gisborne | Lk Waikaremoana | N/A | 2/22/07 |
| *Amphipsalta* | *zelandica* | 07.NZ.KA.KVB.01 | -41.967 | 173.9806 | Kaikoura | NNW of Kekerengu | 105 | 2/17/07 |
| *Amphipsalta* | *zelandica* | 07.NZ.KA.KVB.02 | -41.967 | 173.9806 | Kaikoura | NNW of Kekerengu | 105 | 2/17/07 |
| *Amphipsalta* | *zelandica* | 07.NZ.MB.KOW.02 | -41.714* | 173.112666 | Marlborough | Kowhai Scenic Res., SH63 | N/A | 2/17/07 |
| *Amphipsalta* | *zelandica* | 07.NZ.MB.KOW.04 | -41.714* | 173.112666 | Marlborough | Kowhai Scenic Res., SH63 | N/A | 2/17/07 |
| *Amphipsalta* | *zelandica* | 07.NZ.MB.PVR.01 | -41.508 | 173.51395 | Marlborough | W of Blenheim | 215 | 2/6/07 |
| *Amphipsalta* | *zelandica* | 07.NZ.MB.RAR.01 | -41.789 | 172.9503 | Marlborough | E. of St. Arnaud | 565 | 2/17/07 |
| *Amphipsalta* | *zelandica* | 07.NZ.MB.RAR.03 | -41.789 | 172.9503 | Marlborough | E. of St. Arnaud | 565 | 2/17/07 |
| *Amphipsalta* | *zelandica* | 07.NZ.MC.ASN.01 | -43.901 | 171.753783 | Mid Canterbury | Ashburton | 104 | 2/18/07 |
| *Amphipsalta* | *zelandica* | 07.NZ.MC.OCH.04 | -43.504 | 172.564916 | Mid Canterbury | Christchurch | 29 | 2/18/07 |
| *Amphipsalta* | *zelandica* | 07.NZ.NC.HJC.01 | -42.591 | 172.777283 | North Canterbury | S of Hanmer Springs | 310 | 2/15/07 |
| *Amphipsalta* | *zelandica* | 07.NZ.NC.WUR.12 | -42.658 | 173.029933 | North Canterbury | W. of Waiau | N/A | 2/15/07 |
| *Amphipsalta* | *zelandica* | 07.NZ.NN.OWA.01 | -41.496 | 172.570883 | Nelson | N side of Mt Owen Massif | 1043 | 2/7/07 |
| *Amphipsalta* | *zelandica* | 07.NZ.NN.VWX.01 | -41.613 | 172.666616 | Nelson | 11.3km S of Tui | 417 | 2/8/07 |
| *Amphipsalta* | *zelandica* | 07.NZ.OL.MRV.01 | -44.484 | 168.798133 | Otago Lakes | Matukituki River Vly | 357 | 2/11/07 |
| *Amphipsalta* | *zelandica* | 07.NZ.RI.OLK.01 | -39.433 | 175.377883 | Rangitikei | Ohakune Lake Scenic Res. | N/A | 2/17/07 |
| *Amphipsalta* | *zelandica* | 07.NZ.SC.KAH.01 | -44.157 | 171.055033 | South Canterbury | WSW of Geraldine | 166 | 2/14/07 |
| *Amphipsalta* | *zelandica* | 07.NZ.SC.PPA.02 | -43.889 | 171.26585 | South Canterbury | Peel Forest | 267 | 2/18/07 |
| *Amphipsalta* | *zelandica* | 07.NZ.SL.CLU.02 | -45.926 | 169.482766 | Southland | S. of Beaumont | 48 | 2/12/07 |
| *Amphipsalta* | *zelandica* | 07.NZ.SL.CLU.04 | -45.926 | 169.482766 | Southland | S. of Beaumont | 48 | 2/12/07 |
| *Amphipsalta* | *zelandica* | 07.NZ.SL.CLU.05 | -45.926 | 169.482766 | Southland | S. of Beaumont | 48 | 2/12/07 |
| *Amphipsalta* | *zelandica* | 07.NZ.SL.LIG.02 | -45.791 | 167.647183 | Southland | NW of Blackmount | 146 | 2/20/07 |
| *Amphipsalta* | *zelandica* | 07.NZ.SL.MNP.02 | -45.567 | 167.610683 | Southland | Manapouri | 222 | 2/20/07 |
| *Amphipsalta* | *zelandica* | 07.NZ.SL.MNP.03 | -45.567 | 167.610683 | Southland | Manapouri | 222 | 2/20/07 |
| *Amphipsalta* | *zelandica* | 07.NZ.TK.RWY.04 | -39.058 | 174.057966 | Taranaki | New Plymouth | 20 | 3/1/07 |
| *Amphipsalta* | *zelandica* | 07.NZ.TO.LRP.01 | -39.028 | 175.730666 | Taupo | Lake Rotopounamu | N/A | 2/14/07 |
| *Amphipsalta* | *zelandica* | 07.NZ.TO.PUR.01 | -38.472 | 175.567683 | Taupo | SW of Mangakino | N/A | 2/24/07 |
| *Amphipsalta* | *zelandica* | 07.NZ.TO.PUR.03 | -38.472 | 175.567683 | Taupo | SW of Mangakino | N/A | 2/24/07 |
| *Amphipsalta* | *zelandica* | 07.NZ.TO.PUR.04 | -38.472 | 175.567683 | Taupo | SW of Mangakino | N/A | 2/24/07 |
| *Amphipsalta* | *zelandica* | 07.NZ.TO.RIM.01 | -39.399 | 175.415833 | Taupo | Ohakune Mountain Rd | N/A | 2/16/07 |
| *Amphipsalta* | *zelandica* | 07.NZ.WD.HRB.03 | -43.967 | 169.373033 | Westland | N. of Haast Pass | 104 | 2/10/07 |
| *Amphipsalta* | *zelandica* | 07.NZ.WD.JBZ.01 | -43.874 | 168.985216 | Westland | W. of Haast | 14 | 2/10/07 |
| *Amphipsalta* | *zelandica* | 08.NZ.NN.NGA.01 | -41.202 | 172.887366 | Nelson | 31km North of Tapawera | 61 | 2/2/08 |
| *Amphipsalta* | *zelandica* | 08.NZ.NN.NGA.02 | -41.202 | 172.887366 | Nelson | 31km North of Tapawera | 61 | 2/2/08 |
| *Amphipsalta* | *zelandica* | 08.NZ.NN.NGA.03 | -41.202 | 172.887366 | Nelson | 31km North of Tapawera | 61 | 2/2/08 |
| *Amphipsalta* | *zelandica* | 08.NZ.TH.GRT.01 | -34.1625* | 172.141666 | Three Kings | Great Island | 60 | 11/7/08 |
| *Amphipsalta* | *zelandica* | 08.NZ.TH.GRT.02 | -34.1625* | 172.141666 | Three Kings | Great Island | 60 | 11/7/08 |
| *Amphipsalta* | *zelandica* | 08.NZ.TH.GRT.03 | -34.1625* | 172.141666 | Three Kings | Great Island | 60 | 11/8/08 |
| *Amphipsalta* | *zelandica* | 09.NZ.ND.PKI.01 | -35.463 | 174.738166 | Northland | Poor Knights Is. | N/A | 12/18/09 |
| *Amphipsalta* | *zelandica* | 10.NZ.ND.WHK.01 | -34.948 | 173.544733 | Northland | Whakaangi (TB loc. TB300) | 222 | 1/18/10 |
| *Amphipsalta* | *zelandica* | 94.NZ.WN.APU.01 | -41.304166* | 174.761666 | Wellington | Apuka St., Wellington | N/A | 2/16/94 |
| *Amphipsalta* | *zelandica* | 94.NZ.WN.APU.02 | -41.304166* | 174.761666 | Wellington | Apuka St., Wellington | N/A | 2/16/94 |
| *Amphipsalta* | *zelandica* | 94.NZ.WN.APU.03 | -41.304166* | 174.761666 | Wellington | Apuka St., Wellington | N/A | 2/16/94 |
| *Amphipsalta* | *zelandica* | 94.NZ.WN.BUT.08 | -41.30315* | 174.894733 | Wellington | Eastbourne | 77 | 2/6/94 |
| *Amphipsalta* | *zelandica* | 94.NZ.WN.BUT.09 | -41.30315* | 174.894733 | Wellington | Eastbourne | 77 | 2/6/94 |
| *Amphipsalta* | *zelandica* | 94.NZ.WN.BUT.10 | -41.30315* | 174.894733 | Wellington | Eastbourne | 77 | 2/6/94 |
| *Amphipsalta* | *zelandica* | 96.NZ.KA.URE.01 | -41.906 | 174.1109 | Kaikoura | S. of Ward | 14 | 3/2/96 |
| *Amphipsalta* | *zelandica* | 96.NZ.WN.CRE.01 | -41.281666* | 174.755 | Wellington | Wellington | N/A | 2/19/96 |
| *Amphipsalta* | *zelandica* | 98.NZ.WN.JOH.01 | -41.280583* | 174.741616 | Wellington | Johnston’s Hill, Karori | 225 |  |
| *Amphipsalta* | *zelandica* | 99.NZ.KA.FYF.01 | -42.324 | 173.59755 | Kaikoura | Mt. Fyffe, Kaikoura | 1158 | 3/3/99 |
| *Amphipsalta* | *zelandica* | 99.NZ.KA.FYF.02 | -42.324 | 173.59755 | Kaikoura | Mt. Fyffe, Kaikoura | 1158 | 3/3/99 |
| *Amphipsalta* | *zelandica* | 99.NZ.KA.FYF.03 | -42.324 | 173.59755 | Kaikoura | Mt. Fyffe, Kaikoura | 1158 | 3/3/99 |
| *Amphipsalta* | *zelandica* | 99.NZ.KA.PPU.09 | -42.266 | 173.740516 | Kaikoura | Puhipuhi Reserve | 207 | 3/4/99 |
| *Notopsalta* | *sericea* | 00.NZ.CL.SQT.54 | -36.992 | 175.583333 | Coromandel | NNE of Thornton Bay | N/A | 2/8/00 |
| *Notopsalta* | *sericea* | 01.NZ.TO.WTW.01 | -38.356 | 176.366633 | Taupo | 30 km S. of Rotorua | 343 | 2/2/01 |
| *Notopsalta* | *sericea* | 01.NZ.WA.BUL.03 | -41.323 | 175.304166 | Wairarapa | Bull Hill (Firnan property) | 350 | 12/15/01 |
| *Notopsalta* | *sericea* | 01.NZ.WA.MOI.01 | -41.172 | 175.451666 | Wairarapa | N. of Martinsborough | N/A | 1/21/01 |
| *Notopsalta* | *sericea* | 01.NZ.WO.WGL.01 | -38.262 | 175.104183 | Waikato | Waitomo Caves | 28 | 2/3/01 |
| *Notopsalta* | *sericea* | 02.NZ.BP.ROT.03 | -38.059 | 176.643866 | Bay of Plenty | NW of Kawerau | 187 | 3/5/02 |
| *Notopsalta* | *sericea* | 02.NZ.GB.NWA.02 | -38.894 | 177.262383 | Gisborne | SE of Omahanui | 82 | 12/14/02 |
| *Notopsalta* | *sericea* | 02.NZ.HB.BUR.01 | -39.769 | 176.694433 | Hawke’s Bay | S. of Hastings | 48 | 1/3/02 |
| *Notopsalta* | *sericea* | 02.NZ.HB.BUR.02 | -39.769 | 176.694433 | Hawke’s Bay | S. of Hastings | 48 | 1/3/02 |
| *Notopsalta* | *sericea* | 02.NZ.HB.CAB.04 | -40.407 | 176.530866 | Hawke’s Bay | NE of Wimbleton | 138 | 1/3/02 |
| *Notopsalta* | *sericea* | 02.NZ.HB.MAT.02 | -39.149 | 176.9617 | Hawke’s Bay | SW of Putorino | 173 | 12/13/02 |
| *Notopsalta* | *sericea* | 02.NZ.ND.TOK.02 | -36.061 | 173.967133 | Northland | Tokatoka | 21 | 3/10/02 |
| *Notopsalta* | *sericea* | 02.NZ.WA.CPD.01 | -40.899 | 176.2215 | Wairarapa | Castle Point | 14 | 12/10/02 |
| *Notopsalta* | *sericea* | 03.NZ.BP.WKW.03 | -37.681 | 177.736566 | Bay of Plenty | S. of Waikawa Point | 40 | 3/22/03 |
| *Notopsalta* | *sericea* | 03.NZ.ND.IMM.03 | -35.004 | 173.190766 | Northland | N. of Awanui | 51 | 3/21/03 |
| *Notopsalta* | *sericea* | 03.NZ.WO.PWK.04 | -37.399 | 174.7104 | Waikato | Sunset Beach, Port Waikato | 24 | 3/18/03 |
| *Notopsalta* | *sericea* | 03.NZ.WO.WTS.01 | -38.263 | 175.097 | Waikato | W. of Waitomo | 61 | 3/17/03 |
| *Notopsalta* | *sericea* | 05.NZ.AK.TAP.02 | -36.377 | 174.290166 | Auckland | 20km SW of Wellsford | 4 | 12/18/05 |
| *Notopsalta* | *sericea* | 05.NZ.GB.TRY.03 | -37.865 | 178.18645 | Gisborne | NW of Ruatoria | 123 | 3/8/05 |
| *Notopsalta* | *sericea* | 05.NZ.GB.TWB.02 | -38.856 | 177.794433 | Gisborne | S of Waingake | 621 | 3/9/05 |
| *Notopsalta* | *sericea* | 05.NZ.ND.MAN.01 | -34.993 | 173.5087 | Northland | Mangonui, SH10 | 44 | 3/22/05 |
| *Notopsalta* | *sericea* | 05.NZ.ND.TOB.16 | -34.944 | 173.383583 | Northland | Tokerau Beach | 15 | 3/22/05 |
| *Notopsalta* | *sericea* | 05.NZ.TK.MMN.01 | -38.867 | 174.599483 | Taranaki | N of Mt Messenger on SH3 | 13 | 12/12/05 |
| *Notopsalta* | *sericea* | 05.NZ.TK.MMN.02 | -38.867 | 174.599483 | Taranaki | N of Mt Messenger on SH3 | 13 | 12/12/05 |
| *Notopsalta* | *sericea* | 05.NZ.TO.WRT.04 | -39.023 | 176.563716 | Taupo | NW of Tarawera | 453 | 12/13/05 |
| *Notopsalta* | *sericea* | 06.NZ.ND.KAW.03 | -35.370 | 174.138483 | Northland | E of Kawakawa | 13 | 12/13/06 |
| *Notopsalta* | *sericea* | 06.NZ.ND.KEI.01 | -35.219 | 173.991033 | Northland | E of Kerikeri | 23 | 12/14/06 |
| *Notopsalta* | *sericea* | 06.NZ.RI.CLY.01 | -39.679 | 175.33685 | Rangitikei | 53 km NE of Wanganui | 209 | 1/21/06 |
